# Prediction of SMEs’ R&D performances by machine learning for project selection

**DOI:** 10.1038/s41598-023-34684-w

**Published:** 2023-05-10

**Authors:** Hyoung Sun Yoo, Ye Lim Jung, Seung-Pyo Jun

**Affiliations:** 1 Division of Data Analysis, Korea Institute of Science and Technology Information, Seoul, Republic of Korea; 2 Science and Technology Management and Policy, University of Science and Technology, Seoul, Republic of Korea; 3 Data and High Performance Computing Science, University of Science and Technology, Seoul, Republic of Korea

**Keywords:** Socioeconomic scenarios, Information technology, Statistics

## Abstract

To improve the efficiency of government-funded research and development (R&D) programs for small and medium enterprises, it is necessary to make the process of selecting beneficiary firm objective. We aimed to develop machine learning models to predict the performances of individual R&D projects in advance, and to present an objective method that can be utilized in the project selection. We trained our models on data from 1771 R&D projects conducted in South Korea between 2011 and 2015. The models predict the likelihood of R&D success, commercialization, and patent applications within 5 years of project completion. Key factors for predicting the performances include the research period and area, the ratio of subsidy to research budget, the firm’s region and venture certification, and the average debt ratio of the industry. Our models’ precisions were superior to qualitative expert evaluation, and the machine learning rules could be explained theoretically. We presented a methodology for objectively scoring new R&D projects based on their propensity scores of achieving the performances and balancing them with expert evaluation scores. Our methodology is expected to contribute to improving the efficiency of R&D investment by supplementing qualitative expert evaluation and selecting projects with a high probability of success.

## Introduction

The process of selecting and deciding priorities of research and development (R&D) projects is crucially important for efficiently utilizing limited resources^1, 2^. In the case of government-funded R&D programs, which support a large number of projects with an immense budget, there is an even greater emphasis on the efficient allocation of resources, and many funding agencies devote a lot of effort to improving the process for selecting R&D projects^3, 4^. If one is only considering the efficiency of a program, one may select R&D proposals that are anticipated to yield stronger ex-post performance to be the beneficiary projects of the program^5^. In reality, however, the decision-making process of selecting R&D project is complex and requires consideration of various factors^6–8^. Project selection is complicated because it requires the ex-ante prediction of the performance expected to be achieved by implementing each candidate project, despite the uncertainties involved in such prediction^9^. To do so, we need to precisely define the concept of performance or project success and adopt a commonly accepted method of measuring it. We also need to determine, through theoretical analysis or practical experience, which of the multiple input factors will have an impact on a project’s performance^6^. Furthermore, we need to understand the lag time and uncertainties involved in the process by which R&D activity manifests as performance^9^.

Until now, expert evaluation has been widely utilized to help make complex decisions in the R&D project selection process^10, 11^. Expert evaluation has been considered one of the most rational means of decision-making, since they provide reliable evaluations from a group of experts knowledgeable in a specific field^12, 13^. The expert evaluation method is especially useful for evaluating new ideas regarding which there is no other reference data. We focused on public R&D funding for small and medium enterprises (SMEs), and the R&D proposals by SMEs are also evaluated by experts. R&D projects performed by SMEs are more heterogeneous than science-oriented projects and tend to be application/development-oriented projects. Therefore, in practice, it is operationally worthwhile for experts to review whether the R&D grant is approved or not. However, there have also been several observations regarding the limitations of expert evaluation as a reference for R&D project selection. First of all, the presence of various types of bias, including optimism or pessimism bias, cognitive bias, academic bias, and institutional particularism, may influence subjective judgments, leading to unfair or irrational results^14, 15^. Second, since research disciplines are growing more specialized into sub-areas of expertise while at the same time converging in many aspects, it has become challenging to identify experts or organize groups capable of fully understanding and evaluating all proposals^5^. Third, the R&D project selection process is not structured^15^, and the balance of expert evaluations for various criteria must be considered for rational decision-making^16^. Finally, evaluating a large number of proposals consumes a lot of time and cost, and in cases where the given environment fails to provide adequate time, it may impede evaluators from making optimally rational judgments^10, 15, 16^.

More recently, researchers have proposed various data-based methodologies designed to overcome some of these limitations of expert evaluation^15–19^. Most of these new approaches, however, focus on assigning weighted values to the multiple criteria applied to project selection or on systematically and objectively integrating evaluations from multiple experts^20, 21^. Therefore, they have been limited in addressing the fundamental problems of expert evaluation discussed above. Meanwhile, there has been active empirical research on how to identify the various critical success factors (CSFs) that affect the success of R&D projects^22–25^. However, many of these success factors are difficult to quantify objectively, and most of the empirical studies have relied on the Likert scale to measure survey responses from experts^6, 16^. Several studies utilized machine learning (ML) techniques to determine the relation between project attributes and performance^6, 26, 27^, however, there have been few studies focusing on ex-ante predictions of performances of individual R&D proposals from SMEs and applying such predictions to project selection.

Is it possible to predict the performance of individual candidate projects implemented by SMEs for project selection using only objective data and ML models, without relying on the qualitative judgments of experts? Are prediction rules based on ML both theoretically explainable and practical? How effective is it compared to the qualitative method? It is, of course, unlikely that it will be possible to entirely replace the qualitative and intuitive judgments of experts with an exclusively data-based approach. Moreover, the selection and prioritization of projects is not an issue that can be determined based on efficiency alone. Nonetheless, to enhance the efficiency of public efforts to stimulate the technological innovation of SMEs, we need to offer an objective methodology that can supplement the qualitative judgments of experts. In response to this need, we derived new models that predict whether a candidate project will achieve various performance indicators, including R&D success, commercialization, and patent applications, based on ML analysis of data from a large number of previously completed R&D projects. In the process, we analyzed which of the various factors involved, such as the attributes of projects, firms, and market environments, will strongly affect the performances. We also applied an analytic hierarchy process (AHP) survey of related experts to establish the weighted values to be assigned to the performance indicators for project selection. Based on this, we suggested a method for objectively scoring the expected performances of individual candidate projects. Lastly, we applied the methodology to propose practical ways to improve the current selection process of public funded R&D project for SMEs.

## Methods

### Data

In South Korea, firms with average sales of less than 40 to 150 billion KRW (approximately 33 to 125 million USD, depending on the industry) and total assets of less than 500 billion KRW (approximately 417 million USD) are classified as SMEs. The South Korean government has been implementing various R&D subsidy programs to stimulate the technological innovations of SMEs. Among these, we focused on the “SMEs Technological Innovation Development Program” implemented by South Korea’s Ministry of SMEs and Startups. This program was designed to stimulate technological innovation and exports of SMEs by providing SMEs with R&D subsidies. This program is the largest R&D subsidy program for SMEs in South Korea, with an annual budget of 220 million USD. Each year, the program supported around 500 new firms, and once selected as a beneficiary firm, each firm could usually receive up to 450 to 550 thousand USD over two years. Since this program gives us access to data on many cases of R&D projects, we judged it to be the most suitable for applying our ML technique.

Data on all R&D projects conducted with support from the South Korean government are collected in the National Science & Technology Information Service (NTIS). For each project, NTIS collects around 400 fields of data related to issues such as research period and area, collaborative research, budget and personnel composition, and performances. The data is collected through an annual survey of firms that have implemented R&D, under the supervision of the funding agency. All R&D projects that received public funding must mandatorily submit information regarding the project and its performance to NTIS. NTIS also collects data on performance generated after project completion with lag time. In this study, we collected and utilized NTIS data on 1,771 projects initiated from 2011 to 2013 through the program. Projects begun in 2013 lasted, at maximum, up to 2015. The performance data which was collected up to 2020 (covering a period of five years following project completion) were used to measure the performance indicators.

The attributes of the firm that will perform a project, especially its financial attributes, are resources that could affect the process and outcomes of the project^22^. Accordingly, we used data regarding firm attributes that existed prior to implementing a project as additional attributes data. The market environments in which firms are belonged may also influence the commercialization performance generated by the project^23^. Therefore, we used data on market environments such as market size and competition as additional attributes data. Data on the firm attributes and market environments were obtained from a Korean credit rating agency.

### Variables

If an R&D project is completed normally, without being abandoned or disqualified, an expert committee organized by the funding agency evaluates its performance. The committees give scores and ratings indicating whether projects achieved the technological level set as the target within the given time and budget. Projects that earn a score of 60 or higher are classified as successful R&D projects. We selected the R&D success as one of our performance indicators. In addition, we selected the variable of whether sales were generated from innovative products that applied the developed technology within five years following project completion as the representative indicator of commercialization success. Since the ultimate purpose of a firm’s pursuit of R&D is to generate revenues and profit through successful commercialization, we considered this to be an important criterion of project success. Moreover, we chose the variable of whether a firm applied for a patent within five years of project completion as one of our performance indicators, since it is most closely related to commercialization and the lag time is relatively short.

In this study, we comprehensively considered as many CSFs as possible that could be measured objectively, as well as factors that were not considered in previous studies. We could obtain around 400 features regarding the attributes of projects, firms, and market environments from NTIS and the credit rating agency. First, we screened and eliminated the features that cannot be quantified or input into ML algorithms, such as project titles and research purpose. Then, we reviewed the distribution of each feature and removed the features with excessively skewed distributions. For instance, when dealing with categorical features, we excluded any feature where the majority of cases belonged to a single class as it would not be suitable for classification. Finally, we considered the similarity and multicollinearity among features, and selected representative features. As a result, 41 factor variables were finally selected. Supplementary Material A shows the operational definitions and descriptive statistics of the variables.

Aspects of a project’s scale, including research period, budget, and personnel have important effects on its performance^24, 28^. Moreover, many studies have reported that collaborative research (CR) has a significant influence on firms’ R&D performance^24^. Within NTIS, projects are classified according to various classification systems, based on the characteristics or category of the project; this gives us a supplementary means of judging the contents of projects and grouping them. The size of the firm performing the R&D project, the firm’s age, region, and its area of business are also known to be factors that have a significant effect on performance^3, 22^. In addition, there have been studies indicating that a firm’s financial strength has a positive correlation with R&D intensity and performance^29^. The South Korean government grants venture certification to firms that have received investment above a certain level from venture capital. Moreover, Innobiz certification is granted to firms that are judged to have innovative technologies. Although the process of certification considers a firm comprehensively from various aspects, whether the firm has these certifications at the time of submitting the R&D proposal is objective and can be officially verified. Therefore, we used whether a firm had those certifications as one of the firm attributes. A project’s risks and likelihood of success may also vary depending on the industry^1^. Moreover, market environment factors such as market size, and intensity of competition have also been found to be important factors affecting the success of R&D projects^23^.

### Methodology

Since we considered many factors, we prioritized the use of ML algorithms that are better suited for identifying complex relationships among multiple variables. Since the performance indicators are binary variables, we used various classification algorithms. There is a wide variety of classification algorithms, each with its own pros and cons; there is no single best algorithm that is superior to all other models and applicable to all cases^30^. Therefore, after comparing the performances of models generated using various algorithms, we selected the model that demonstrated the strongest prediction performance as an optimal model. The ML algorithms used in this study included rule-based Decision Tree (DT) such as Classification and Regression Tree (CART), C5.0, Chi-squared Automatic Interaction Detector (CHAID), Quick, Unbiased, Efficient Statistical Tree (QUEST), Random Forest (RF), and non-linear algorithms Neural Network (NN) and Support Vector Machine (SVM). We also compared our results with those obtained using conventional linear algorithms such as Logistic Regression (LR) and Discriminant Analysis (DA). Through this process, we compared the prediction performances of the ML algorithms with those of linear algorithms. Moreover, we compared the rules derived by ML with those by linear algorithms, and interpreted them theoretically. Supplementary Material B provides details on the characteristics, strengths, and weaknesses of each algorithm we used.

To apply such classification algorithms, we used IBM’s SPSS Modeler 18. We divided the data at a ratio of 7:3 into training data and test data. If the dependent variables’ group distribution is overly skewed to one side, there is a strong likelihood that the algorithm will classify most of the cases as belonging to the majority group, just to obtain high accuracy. To prevent this, we used the bootstrapping method to balance the training data’s distribution to be 5:5. In the case of DT and NN, in which results vary depending on the cross-validation data set for preventing overfitting, we generated 100 models with each algorithm and performed bagging. Parameter tuning was performed to minimize overfitting for each ML algorithm. In DA, we used the method of adding or eliminating variables that minimize Wilks' lambda at each stage to select the key factors. In LR analysis, we selected key factors using a forward stepwise method based on the likelihood ratio. The ML prediction models for a binary dependent variable provide raw propensity scores (RPS) for classification. These scores not only give us insight regarding whether each case will be grouped as true or false but also inform us of the probability value of the prediction. They allow us to predict the feasibility of the three kinds of performances for each candidate project. We conducted an AHP survey to determine which of the three performance indicators should be given greater weight when selecting the beneficiary firms of the program. 21 experts, who participated in the program’s project planning and evaluation, responded to our survey. All 21 experts have a doctorate degree, in various scholarly fields, and are currently engaged in R&D planning and evaluation for SMEs and policy research. Supplementary Material C shows the demographics of the 21 experts.

## Results and discussion

### Machine learning models for prediction of SME’s R&D performances

Table 1 presents the classification performances of the prediction models for the three performance indicators using each ML and linear algorithm. We basically performed a comparison of the classification accuracy and also presented the values of precision, recall, and F-measure. In this study, we assumed a scenario in which firms predicted to achieve significant performance are properly discriminated and assigned additional points, which increase the likelihood of these firms being chosen as beneficiary firms. Therefore, for the binary performance indicators, we compared the precision, recall, and F-measure based on the group that is predicted to achieve the performance (i.e., “true”).

**Table 1:** Classification performances of the prediction models.

Performance indicators	Algorithms	Accuracy (%)		Precision	Recall	F-measure
		Training	Test	Test	Test	Test
R&D success	CART	82.5	72.4	0.935	0.735	0.823
	CHAID	92.4	75.7	0.905	0.806	0.853
	C5.0	97.9	84.5	0.905	0.806	0.912
	QUEST	79.4	75.7	0.915	0.795	0.851
	RF	89.3	81.2	0.878	0.911	0.894
	NN	84.9	70.0	0.895	0.742	0.811
	SVM	90.7	77.9	0.910	0.829	0.867
	LR	73.1	72.6	0.916	0.755	0.828
	DA	66.0	64.7	0.903	0.666	0.767
Commercialization	CART	73.8	68.0	0.692	0.945	0.799
	CHAID	74.7	67.4	0.689	0.939	0.795
	C5.0	72.5	71.3	0.740	0.885	0.806
	QUEST	68.8	67.2	0.689	0.937	0.794
	RF	92.8	69.5	0.712	0.919	0.803
	NN	67.3	64.1	0.686	0.862	0.764
	SVM	73.3	67.2	0.725	0.827	0.773
	LR	66.6	66.6	0.694	0.902	0.784
	DA	63.5	62.3	0.673	0.856	0.754
Patent applications	CART	78.9	59.8	0.621	0.689	0.653
	CHAID	77.5	59.4	0.614	0.703	0.656
	C5.0	65.6	63.9	0.637	0.799	0.708
	QUEST	62.1	61.9	0.614	0.827	0.705
	RF	93.8	61.4	0.627	0.731	0.675
	NN	73.5	59.2	0.598	0.788	0.680
	SVM	80.1	62.7	0.653	0.686	0.669
	LR	58.9	56.3	0.593	0.654	0.622
	DA	60.1	58.4	0.618	0.640	0.628

In terms of accuracy and F-measure in the test data, the prediction models by C5.0 algorithm showed the highest classification performance for all performance indicators. The prediction model for R&D success demonstrated classification accuracies of 97.9% for the training data and 84.5% for the test data. This model also yielded an F-measure of 0.912, demonstrating strong performance in classifying the groups found to be “true” in regard to R&D success. The classification accuracy in the test data of the optimal prediction model for commercialization was 71.3%, and the F-measure was 0.806. For patent applications, classification accuracy and F-measure were 63.9% and 0.708, respectively.

Some of the 41 factors played an important role in predicting the performances. Table 2 shows the key factors for each performance indicator by the optimal C5.0 models. In the case of R&D success, the key factors were venture certification, assets, application area, ratio of subsidy, and number of CR in that order. In the case of commercialization, the key factors include research period, venture certification, Innobiz certification, average debt ratio, and firm age. To predict patent applications, research period, venture certification, located in metropolitan, research budget, and ratio of MS & Ph.D. researchers played the most important role. There were factors that played an important role in common for all performance indicators. Among the project attributes, research period, research area, ratio of subsidy, and ratio of MS & Ph.D. researchers were important. Moreover, among the firm attributes, venture certification and located in metropolitan were important variables in common, and among the market environments, the average debt ratio of the industry was important.

**Table 2:** Key factors for prediction of the R&D performances by the optimal C5.0 models.

R&D success		Commercialization		Patent applications	
Key factors	Importance	Key factors	Importance	Key factors	Importance
Venture certification	0.15	Research period	0.18	Research period	0.12
Assets	0.13	Venture certification	0.14	Venture certification	0.12
Application area	0.09	Innobiz certification	0.14	Located in metropolitan	0.10
Ratio of subsidy	0.07	Average debt ratio	0.13	Research budget	0.09
Number of CR	0.06	Firm age	0.09	Ratio of MS & Ph.D. researchers	0.09
Ratio of female researchers	0.06	Located in metropolitan	0.07	Number of CR	0.07
Average debt ratio	0.04	Ratio of subsidy	0.05	Average total asset turnover	0.06
Debt ratio	0.04	Total sales	0.05	Ratio of female researchers	0.05
Ratio of personal cost	0.04	Ratio of budget to sales	0.05	R&D investments	0.05
Located in metropolitan	0.03	Research budget	0.03	Debt ratio	0.05
Return on equity	0.03	Research area	0.03	Ratio of subsidy	0.05
Ratio of MS & Ph.D. researchers	0.03	Ratio of cash	0.03	Budget for CR	0.04
Operating profit ratio	0.03	Industry	0.01	Average debt ratio	0.03
Ratio of researchers with degrees in Eng	0.03	Application area	0.01	Researchers	0.03
Current ratio	0.03			Research area	0.02
Payables turnover	0.03			Innobiz certification	0.01
Ratio of cash	0.03				
Innobiz certification	0.02				
Research period	0.02				
Total assets turnover	0.01				
Research area	0.01				
Average total asset turnover	0.01				
Industry	0.01				

### Comparison with linear models and theoretical interpretation of the rules

As indicated in Table 1, the prediction models derived from the various ML algorithms showed stronger prediction performance compared to conventional linear statistical techniques such as LR and DA. For R&D success, commercialization, and patent applications, the optimal C5.0 models had higher accuracy in the test data compared to the results from DA, by a margin of respectively 19.8%p, 9.0%p, and 5.5%p. Linear algorithms select key variables based on statistical significance tests on their relationship with performance indicators. Therefore, relatively few variables are included in the classification rule. In addition, in the case of DA, only continuous variables are used to generate a classification model. On the other hand, ML algorithms utilize relatively more variables, which is one of the reasons for the higher performance.

We generated 100 models for each ML algorithm and performed bagging. Among the models generated by the C5.0 algorithm, representative models that appear repeatedly are shown in Supplementary Material D. Each rule by C5.0 can be theoretically explained in connection with the results of preceding studies. In the rule for R&D success, firms that were pre-certified by venture capital or public agencies as having innovative potential and capacity were found to be more likely to achieve R&D success. In addition, compared to projects with a research period of 1 year, projects with relatively sufficient time (2 years) were more likely to achieve R&D success^24^. A firm's assets can act as an important resource and capability to continuously and stably carry out R&D^29^. Firms with a high debt ratio or firms that belong to an industry with a high average debt ratio, resulting in lower financial stability, were notably found to have relative weak likelihood of R&D success. It was found that there is a difference in the likelihood of R&D success depending on research area, application area, and industry to which the firm belongs, because the process and difficulty of R&D are different^1^. Regarding the composition of research budget, it can be understood that the higher the ratio of cash with a high degree of freedom in use, the more effective research is promoted, which contributes to R&D success. In addition, the lower the ratio of subsidy by increasing the firm's own contribution, the more active and responsible for R&D, the higher the likelihood of R&D success.

In terms of commercialization, firms that had both venture and Innobiz certifications were found to have a higher likelihood of success. Sufficient research period was found to have a positive effect on commercialization as well^24^. Meanwhile, firm age was found to be a negative factor affecting commercialization. It can be attributed to the fact that firms that have operated well for at least a certain number of years tend to have a significant proportion of its production capabilities already devoted to an existing flagship product, which may delay the timing of input for new products and delay sales^31^. It was found that there is a difference in the commercialization process depending on research area, and thus there is a difference in the likelihood of commercialization success^22^. Firms in industries with low average debt ratios could be more likely to succeed in commercialization^29^. In addition, factories of large corporations in South Korea's major industries are not mainly located in the metropolitan area, and most of the major SMEs that supply parts and equipment to the factories are also the same. It is understood that SMEs located close to the factories of large corporations increase their chances of success based on more information and opportunities for commercialization.

As with other performance indicators, venture certification, Innobiz certification, and research period were found to have a positive effect on patent applications. In addition, firms with a low ratio of subsidy due to their high contribution and active involvement were found to have relatively high patent application performance. We found that a higher ratio of researchers with MS & Ph.D. degrees raised the possibility of patent applications. To apply a patent, it is necessary to draw on in-depth knowledge of cutting-edge technologies to persuasively demonstrate novelty and progress, and therefore, participating researchers with more experiences in related fields will increase the possibility of achieving patents^24^. It was found that patent applications of SMEs differed depending on research area^22^. Firms with a small debt ratio, high average total asset turnover, and low average debt ratio were more likely to apply for a patent^29^. Ratio of female researchers is one of the key factors that positively affect R&D success and patent applications. The majority (> 85%) of researchers belonging to South Korean SMEs are male, and gender diversity is very low^32^. An increase in the ratio of female researchers to a certain level (i.e., improving gender diversity) in the male-dominated teams could have a positive impact on the performances by providing a variety of perspectives, ways of thinking, and sources of information^33^.

Supplementary Material E shows the LR models on each performance indicator. Although the linear models had somewhat inferior classification performance, they provided more concise rules that were statistically significant. The variables selected based on the statistical significance for each performance indicator are well included in the key factors by ML shown in Table 2. The direction of the effect of key factors selected in the LR models on each performance indicator was also in good agreement with the optimal rules by the ML algorithm. The rules by ML can cover the rules by statistically significant linear models, provide better prediction performance, and can be explained sufficiently theoretically.

### Objective prediction and its use in project selection

**Figure 1:**
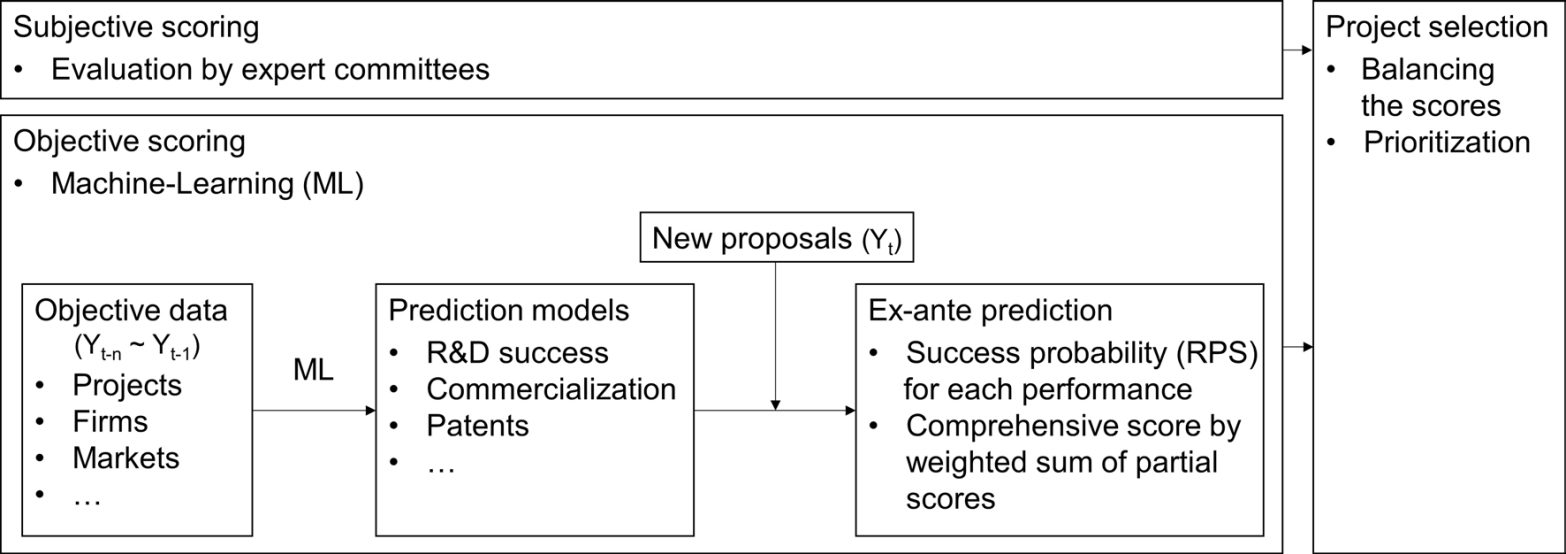
Project selection process applying the objective prediction results for R&D performances.

This study established the prediction models for three performance indicators to objectively predict the performances of newly proposed projects and presents a method of integrating and scoring these results. Figure 1 shows how the objective prediction of performances by ML is utilized in project selection process in harmony with the qualitative evaluation of expert committees.

According to the AHP survey results, commercialization is the most important aspect of expected performance, and its relative magnitude of importance was derived to be 0.514. This is understandable if we consider that the ultimate purpose of SMEs undertaking R&D is to gain revenues and profit from innovative products that apply new technologies. This was followed in order by the possibility of R&D success (0.366) and patent applications (0.120). The consistency ratio was 0.007, indicating that the survey had achieved consistency. Table 3 shows the process by which we predicted the performances and deduced the comprehensive scores of ten projects that newly received the program’s support in 2014. We used the optimal models respectively found to have the strongest performances. For each of the ten new candidate projects, we derived the predicted group for the three performance indicators and the RPS. RPS compares the probability of each candidate project achieving the performances after receiving subsidies under the same conditions. We used RPS to calculate the partial score. As mentioned earlier, our goal is to assign additional points to projects that have a higher probability of success as an outcome of receiving the same support. On the other hand, if the machine predicts that someone's R&D plans will fail to achieve the performances, we should be very careful in accepting it. In cases where a candidate project was predicted to achieve the performances, we applied the RPS directly as the partial score but in cases where this was not true, we assigned the basic score of 0.5 points. Then, we also applied the weighted values for each performance indicator, and calculated the comprehensive score using the weighted sum of the partial scores. This demonstrates that it is possible to objectively and comprehensively score performances using only objectively measurable data and rules derived by ML, while completely excluding the subjective judgments of experts.

**Table 3:** Deduction of the comprehensive scores of ten candidate projects.

Performance indicators	R&D success weight: 0.366			Commercialization weight: 0.514			Patent applications weight: 0.120			Comprehensive scores
Candidates	Pred. group	RPS	Partial score	Pred. group	RPS	Partial score	Pred. group	RPS	Partial score	
1	True	0.725	0.725	False	0.206	0.500	True	0.695	0.695	0.606
2	True	0.805	0.805	True	0.656	0.656	True	0.754	0.754	0.722
3	True	0.756	0.756	False	0.191	0.500	True	0.811	0.811	0.631
4	False	0.280	0.500	False	0.243	0.500	True	0.766	0.766	0.532
5	True	0.918	0.918	True	0.669	0.669	False	0.229	0.500	0.740
6	True	0.654	0.654	True	0.675	0.675	False	0.362	0.500	0.646
7	True	0.597	0.597	False	0.229	0.500	True	0.740	0.740	0.564
8	True	0.813	0.813	True	0.691	0.691	True	0.613	0.613	0.726
9	True	0.757	0.757	True	0.666	0.666	True	0.669	0.669	0.700
10	True	0.623	0.623	False	0.258	0.500	False	0.365	0.500	0.545

We are not arguing that this objective prediction is perfectly accurate or entirely eradicates bias or that it can completely replace the role of subjective judgments. We aimed to demonstrate its utility as a supplementary method and to prove its superiority to subjective judgments in certain aspects. This study demonstrates the possibility of the objectively scoring based on quantitatively measured data and the objective rules for predicting performances that do not vary depending on the evaluator. The 41 factors listed in Table SA1, are measured objectively, and since their values are already determined, it does not vary depending on the individual performing the measurement. Moreover, the relation between the factors and a performance indicator, which we refer to as a prediction rule, is generated through ML, and since we choose the optimal rules with the strongest prediction performance among those obtained through various algorithms, such rules will not vary depending on who the evaluator may be. Of course, even objectively measured and obtained data may be biased depending on how it is sampled. However, our analysis included all projects supported by the R&D subsidy program and we generated rules applicable to all research fields. Therefore, we believe that it is possible to significantly reduce the various types of bias that may appear in the qualitative judgments of experts in the objective scoring process.

The prediction performance can also be superior compared to that based on subjective judgments. All 1,771 projects that benefited from the program were selected because they were predicted to yield strong performance, based on the evaluation of expert committees. Therefore, the projects’ performance can be interpreted as the precision (TP/P) of the expert committees, and the precisions for R&D success, commercialization, and patent applications were respectively 85%, 65%, and 52%. By contrast, as shown in Table 1, the precisions of our optimal ML models were respectively 91%, 74%, and 64%, superior to the precision from the qualitative judgments of experts. This demonstrates that an entirely objective method is clearly not inferior in prediction performance compared to the current method relying on experts’ qualitative judgments and the objective method enables the selection of projects with a higher probability of success, thereby improving the program’s efficiency.

However, machines cannot entirely replace the role of people. The results generated by machines should be used only as a means to supplement the qualitative judgments of experts. The existing selection process relied only on the qualitative evaluation by experts, and the expert's score accounted for 100% of the score for selecting beneficiary firms. The objective evaluation method proposed in this study can be conducted independently of the experts’ evaluation. We suggest that a composite score can be derived by giving appropriate weights (for example, 70%:30%) to the expert's qualitative score and the machine's objective score. Rather than relying solely on the qualitative scores from experts, the composite score can be used to prioritize new R&D proposals and select beneficiaries. In this way, the qualitative judgment of experts could be supplemented, and the existing subjective evaluation system can be objectified to a certain extent.

## Conclusion

Efficiency should not be the only criterion considered for R&D project selection and budget allocation. In addition to efficiency, diversity and urgency should be considered as well. Nonetheless, improving efficiency is a challenge currently confronted by governmental R&D funding agencies in many countries. To improve the efficiency, we aimed to develop ML models to predict the performances of individual R&D projects in advance, and to present an objective method that can be utilized in the project selection.

The key findings and contributions of this study are as follows. First, from a theoretical perspective, we derived key factors that can influence performances that SMEs can achieve through R&D, such as R&D success, commercialization, and patent applications, using ML and linear algorithms. We showed how each key factor affects the performances through the explainable rules derived from the algorithms. In addition, we provided additional empirical evidence for the relationships between them and theoretically interpret the relationships. Moreover, we showed that the relationships are in good agreement with the results of previous studies and theoretically explainable. For practical application, we provide a method for the project selection process that overcomes the limitations posed by previous reliance on experts’ evaluations, and thereby promotes greater efficiency in the execution of public R&D funding. Different from other studies, this study proved that it is possible to use only objectively measured data on 41 factors and objective rules derived from ML to perform ex-ante predictions of the performances. We also demonstrated that the objective method can perform better than the qualitative expert evaluation. This study is a case study that target R&D projects implemented by South Korean SMEs with government subsidies. However, data on R&D projects are rapidly accumulating in many countries, and the methodology presented in this study can be applied sufficiently. In methodological perspective, by using ML algorithms, this study was able to take account of a larger number of factors more flexibly and comprehensively, compared to studies that used conventional econometric models. This study demonstrates that it is possible to perform comprehensive scoring using the RPS for 3 performance indicators, and a significant contribution of this study is that it offers a method of using this scoring in the project selection process.

This study has several limitations. First, the models we developed have room for further improvement in predictive performance. We mainly used relatively simple DT-based algorithms to derive explainable rules and theoretically investigate the effect of each key factor. Further studies applying more advanced algorithms such as multitask learning and transfer learning would also be meaningful in the future for performance improvement. Of course, the efforts to include larger volumes of data and add more important variables in the models are also important. The efforts to empirically validate the methodology proposed in this study are also required. Efforts should continue to find and improve the limitations of ML methodologies by applying them to various R&D subsidy programs in many countries. Moreover, it is also important to find ways to utilize the methodology in individual firms as a follow-up study. According to a recent study, corporate R&D investment decision makers tend to have higher trust in AI-based advisory systems than human advisors^34^. If the methodology were to be provided in the form of web services with public statistical data that can serve as a data source for various factors, we anticipate that individual firms will also be able to perform their own evaluations in the R&D planning stage. This can be done without the help of experts, to predict performance and identify ways to maximize it.

## Supplementary Information

Supplementary Information.

## Data Availability

The data that support the findings of this study are available from the corresponding author upon reasonable request.
