# Supplementary material for: Prediction of SMEs’ R&D performances by machine learning for project selection
Source: Sci Rep. 2023 May 10;13:7598. doi: 10.1038/s41598-023-34684-w (PMC10172173; doi:10.1038/s41598-023-34684-w)
Supplement: Supplementary file 1 — Supplementary Information. [file 41598_2023_34684_MOESM1_ESM.pdf]

Supplementary Materials

Prediction of SMEs' R&D Performances by Machine Learning for Project Selection

Hyoung Sun Yoo[1, 2,*], Ye Lim Jung[1, 3], and Seung-Pyo Jun[1, 2]

[1]Division of Data Analysis, Korea Institute of Science and Technology Information, Seoul, Rep. of Korea.

[2] Science & Technology Management & Policy, University of Science & Technology, Seoul, Rep. of Korea.

[3]Data & High Performance Computing Science, University of Science & Technology, Seoul, Rep. of Korea.

[*]corresponding author e-mail: hsyoo@kisti.re.kr

Supplementary Material A The operational definitions and descriptive statistics of the variables

Table SA1. The operational definitions of the variables

Variables			Explanation
**Performance indicators**	R&D success		Whether the focal R&D project was determined to have succeeded in technology development by an expert evaluation committee
	Commercialization		Whether the firm that implemented the focal R&D project generated sales from innovative products that applied the developed technology within five years following project completion
	Patent applications		Whether the firm that implemented the focal R&D project applied for a patent regarding the developed technology within five years following project completion
**Project attributes**	Period	Research period	The total period of the focal R&D project
	Budget	Research budget	The total budget spent on the focal R&D project
		Ratio of subsidy	The share of government subsidy in the total R&D budget
		Ratio of personnel cost	The share of personnel cost in the total R&D budget
		Ratio of cash	The cash share of the total R&D budget
	Personnel	Researchers	The total number of researchers involved in the focal R&D project
		Ratio of female researchers	The ratio of female researchers to the total number of researchers
		Ratio of MS & Ph.D. researchers	The ratio of MS & Ph.D. researchers to the total number of researchers
		Ratio of researchers with degrees in Eng.	The ratio of researchers with degrees in engineering to the total number of researchers
	Collaborative research (CR)	Number of CR	The number of CR projects conducted as parts of the focal R&D project
		Budget for CR	The research budget allocated to the CR projects
		Ratio of budget for CR	The ratio of budget for CR projects to the total R&D budget
	Area	Research area	The research areas based on the Korean National Science and Technology Standard Classification
		Application area	The application areas based on the Korean National Science and Technology Standard Classification
		6T classification	The classification according to the 6T classification of the technology to be developed through the focal R&D project
**Firm attributes**	Basic attributes	Industry	The industry in which the focal firm belongs based on the Korea Standard Industry Classification
		Firm age	The age of the focal firm one year before the initiation of its R&D project
		Located in metropolitan	Whether the focal firm's headquarters is located in a metropolitan area
		Venture certification	Whether the focal firm has secured the venture certification
		Innobiz certification	Whether the focal firm has secured the Innobiz certification
	Finance	Sales	The sales of the focal firm one year before the initiation of its R&D project
		Assets	The total assets of the focal firm one year before the initiation of its R&D project
		Operating profit ratio	The operating profit ratio of the focal firm one year before the initiation of its R&D project

		Return on assets	The return on assets of the focal firm one year before the initiation of its R&D project
		Return on equity	The return on equity of the focal firm one year before the initiation of its R&D project
		Current ratio	The current ratio of the focal firm one year before the initiation of its R&D project
		Debt ratio	The debt ratio of the focal firm one year before the initiation of its R&D project
		Inventory turnover	The inventory turnover of the focal firm one year before the initiation of its R&D project
		Receivables turnover	The receivables turnover of the focal firm one year before the initiation of its R&D project
		Payables turnover	The payables turnover of the focal firm one year before the initiation of its R&D project
		Total asset turnover	The total asset turnover of the focal firm one year before the initiation of its R&D project
		R&D investments	The R&D investments of the focal firm one year before the initiation of its R&D project
		Ratio of R&D investment to sales	The R&D intensity of the focal firm one year before the initiation of its R&D project
		Ratio of budget to sales	The ratio of the total R&D budget to sales of the focal firm one year before the initiation of its R&D project
**Market environments**	Competition	Number of competitors	The number of firms competing in the industry in which the focal firm belongs
		Market concentration	The market concentration measured by Herfindal-Hershman Index (Sum of square of market share of each firm)
	Industry finance	Total sales	The total sales of firms in the industry in which the focal firm belongs
		Total assets	The total assets of firms in the industry in which the focal firm belongs
		Average operating profit ratio	The average operating profit ratio of firms in the industry in which the focal firm belongs
		Average debt ratio	The average debt ratio of firms in the industry in which the focal firm belongs
		Average total asset turnover	The average total asset turnover of firms in the industry in which the focal firm belongs

Table SA2. The descriptive statistics of the variables

Variables			Unit	Min.	Max.	Mean	S.D.
**Performance indicators**	R&D success		True/False (85:15)				
	Commercialization		True/False (65:35)				
	Patent applications		True/False (52:47)				
**Project attributes**	Period	Research period	years	1	2	1.77	0.42
	Budget	Research budget	T USD	60.60	1589.73	477.97	253.60
		Ratio of subsidy	-	0.34	0.94	0.75	0.09
		Ratio of personnel cost	-	0.00	0.98	0.33	0.19
		Ratio of cash	-	0.55	1.00	0.97	0.06
	Personnel	Researchers	persons	1	73	16.22	10.60
		Ratio of female researchers	-	0.00	1.00	0.14	0.16
		Ratio of MS & Ph.D. researchers	-	0.00	1.00	0.39	0.26
		Ratio of researchers with degrees in Eng.	-	0.00	1.00	0.70	0.27
	Collaborative research (CR)	Number of CR	cases	0	4	1.02	1.07
		Budget for CR	T USD	0	364	47	57
		Ratio of budget for CR	-	0	0.36	0.09	0.10
	Area	Research area	7 classes (e.g. Chemistry)				
		Application area	10 classes (e.g. Machinery)				
		6T classification	7 classes (e.g. Information Technology)				
**Firm attributes**	Basic attributes	Industry	39 classes (ex. Agriculture)				
		Firm age	year	0	64	10.48	7.80
		Located in metropolitan	True/False (70:30)				
		Venture certification	True/False (38:62)				
		Innobiz certification	True/False (46:54)				
	Finance	Sales	T USD	0	223,537	12,700	20,680
		Assets	T USD	0.28	99,833	4,781	9,227
		Operating profit ratio	-	-192.14	0.45	-0.47	8.05
		Return on assets	-	-2.55	1.06	0.04	0.19
		Return on equity	-	-33.21	15.35	0.06	1.17
		Current ratio	-	0.06	1349.01	3.98	36.65
		Debt ratio	-	-20.99	215.72	1.79	6.03
		Inventory turnover	times	0.01	20639	4058	8158
		Receivables turnover	times	0.09	6955	397	1579
		Payables turnover	times	0.02	118075	16931	41293
		Total asset turnover	times	0.00	8.57	1.18	0.82
		R&D investments	T USD	0.00	14,528	438	838
		Ratio of R&D investment to sales	-	0.00	85.44	0.21	2.72
		Ratio of budget to sales	-	0.00	123.11	0.62	5.14
**Market environments**	Competition	Number of competitors	firms	1	4,577	776	1,225
		Market concentration	-	0.00	1.00	0.09	0.13

	Industry finance	Total sales	M USD	5	155,104	7,119	12,093
		Total assets	M USD	3	144,610	6,185	10,462
		Average operating profit ratio	-	-0.17	0.35	0.05	0.04
		Average debt ratio	-	-13.67	9.58	1.18	0.75
		Average total asset turnover	times	0.04	16.06	1.14	0.50

Supplementary Material B The classification algorithms used in this study

Although a wide variety of classification algorithms are currently in use, leading examples are conventional linear algorithms such as LR (Logistic Regression) and DA (Discriminant Analysis), non-linear algorithms such as NN (Neural Network) and SVM (Support Vector Machine), and rule-based DT (Decision Trees). Linear algorithms adopt a mathematical approach to determine a group of a dependent variable from input variables; that is, they generate a linear function to determine a group. By contrast, non-linear algorithms generate more complex non-linear functions. Rule-based algorithms, in contrast to those that perform scoring or splitting by inputting certain input data into a function to predict a group, use the input data itself as the basis for identifying the hieratical rules that can best divide groups. In each stage of the rules, the case node is split, and this node is determined as the group that minimizes misclassification. Linear algorithms consist of a parametric method, which requires the presumption of a Gaussian distribution in the data and the strict processing of outliers and missing values, but they operate well on small case data and are generally robust, allowing us to judge the statistical significance of a model. Non-linear algorithms work reliably even on data with many variables and many cases, as well as on data consisting of multiple types and distributions. However, they pose some challenging issues, such as the difficulty of understanding their rules and results and the problem of overfitting and local optima. Rule-based DT is free in terms of data distribution presumptions and operate well on data containing many variables and many cases, with the advantage that the rules and results are explainable. However, rule-based DT also require caution regarding local optima and overfitting. The pros and cons of each algorithm are summarized in Table SB1.

Table SB1. The characteristics of the classification algorithms

**Classification algorithms**		**Merits**	**Demerits**
**Linear**	**Logistic Regression (LR)**	- Robust (barely influenced by noise.)<br>- Works with a small case data.<br>- Probabilistic interpretation of results.	- Problems with high collinearity.<br>- Gaussian distributed residuals.<br>- No missing values.
	**Discriminant Analysis (DA)**	- Very fast.<br>- Works with a small case data.<br>- Optimal if data assumptions are fulfilled.	- Only applicable to numeric variables.<br>- Class-wise Gaussian distributed.<br>- Sensitive to outliers.
**Non-Linear**	**Neural Network (NN)**	- Suitable for a lot of case data.<br>- Applicable to very complex. relationships between variables.<br>- No assumptions about distribution of variables are needed.<br>- Resistant to defective data.	- Results and rules are hard to understand.<br>- May fall into local optima.
	**Support Vector Machine (SVM)**	- Robust (barely influenced by noise.)<br>- Applicable to many variables.	- Results and rules are hard to understand.<br>- Sensitive to the choice of kernel function.<br>- Trends to overfit.
**Rule-Based**	**Decision Trees (DT)**	- Results and rules are understandable.<br>- Works with any types of data.<br>- Suitable for a lot of case data.<br>- No assumptions about distribution. of variables are needed.<br>- Not sensitive to outliers.	- Trends to overfit.<br>- May fall into local optima.<br>- Prefers variables with many categories or numerical data.

DT include various detailed algorithms such as the Classification and Regression Tree (CART), C5.0, the Chi-squared Automatic Interaction Detector (CHAID), and the Quick, Unbiased, Efficient Statistical Tree (QUEST). CART is a binary splitting algorithm and provides a pruning function to prevent overfitting. CART uses the Gini coefficient, which expresses how low the impurity is within a single node, to determine the split rules, and identifies the rule that maximizes the Gini Gain, which indicates how much the Gini coefficient increases when split to the next stage. C5.0 can split a node into two or more sub-nodes and provides a pruning function. C5.0 is similar to CART in that it uses the Entropy indicating the heterogeneity within a single node and finds the rule that maximizes the Information Gain, which indicates the decrease in Entropy when split to the next stage. CHAID, like C5.0, can split a node into two or more sub nodes but does not provide a pruning function. CAHID uses the Chi-square test to verify the statistical similarity of a dependent variable according to independent variables and the variable with the highest significance is determined to be the split rule. QUEST, like CART, generates binary trees and provides a pruning function. Like CHAID, it uses the Chi-square test to generate the split rule. The characteristics of these algorithms are shown in Table SB2[1].

Table SB2. The characteristics of the DT algorithms

**DT algorithms**	**CART**	**C5.0**	**CHAID**	**QUEST**
**Target variable type**	Numerical, categorical	Categorical	Numerical, categorical	Categorical
**Input variable type**	Numerical, categorical	Numerical, categorical	Numerical, categorical	Numerical, categorical
**Split criteria**	Gini coefficient	Entropy	Chi-squared statistics	Chi-squared statistics
**Split method**	Binary	2 or more	2 or more	Binary
**Pruning**	Provided	Provided	Not provided	Provided

References

1 Wendler, T. & Gröttrup, S. *Data mining with SPSS modeler: theory, exercises and solutions.* (Springer, 2016).

Supplementary Material C The demographics of the experts who responded to the AHP survey

Table SC1. The demographics of the experts who responded to the AHP survey

Category	Class	Persons
**Gender**	Female	6
	Male	15
	Total	21
**Background**	Bio & Medicine	2
	Chemistry	3
	Economics	1
	Electric & Electronics	1
	Energy & Resources	2
	ICT	2
	Industrial Eng.	2
	Machinery & Materials	2
	Management	4
	Policy	2
	Total	21
**Areas of participation in the subsidy program***	Planning	15
	Evaluation	12
	Policy	11

*An expert can participate in several areas.

Supplementary Material D1 A prediction rule for R&D success by ML (C5.0)

1 Innobiz certification = yes (681)
  1.1 Research period <= 1.5 years (156)
    1.1.1 Industry = Manufacturing of machinary and equipment (69)
      1.1.1.1 Ratio of cash <= 0.995 (32) : False (97.1%)
      1.1.1.2 Ratio of cash > 0.995 (37)
        1.1.1.2.1 Research area = Bio & Medical, ICT (5) : True (100%)
        1.1.1.2.2 Research area = Others (31)
          1.1.1.2.2.1 Debt ratio <= 1.281 (10) : True (65.3%)
          1.1.1.2.2.2 Debt ratio > 1.281 (22) : False (96.8%)
    1.1.2 Industry = Others (87) : True (82.8%)
  1.2 Research period > 1.5 years (525) : True (88.6%)
2. Innobiz cerification = no (1,145)
  2.1 Venture cerification = no (970)
    2.1.1 Assets <= 27,441 T USD (899)
      2.1.1.1 Industry = Manufacture of chemicals and chemical products, Manufacture of rubber and plastics products (170) : True (81.1%)
      2.1.1.2 Industry = Manufacture of electrical equipment (55)
        2.1.1.2.1 Located in metropolitan = no (20) : True (90.3%)
        2.1.1.2.2 Located in metropolitan = yes (35) : False (91.3%)
      2.1.1.3 Industry = Publishing activities (191)
        2.1.1.3.1 Total asset turnover > 2.034 (15) : True (86.4%)
        2.1.1.3.2 Total asset turnover <= 2.034 (176)
          2.1.1.3.2.1 Research area = Knowledge service (17) : True (85.8%)
          2.1.1.3.2.2 Research area = Others (159) : False (82.8%)
      2.1.1.4 Industry = Manufacture of fabricated metal products (21)
        2.1.1.4.1 Number of CR <= 1.5 (13) : False (92.1%)
        2.1.1.4.2 Number of CR > 1.5 (8) : True (100%)
      2.1.1.5 Industry = Manufacture of electronic components, computer (106)
        2.1.1.5.1 Located in metropolitan = no (13) : True (100%)
        2.1.1.5.2 Located in metropolitan = yes (93) : False (79.9%)
      2.1.1.6 Industry = Others (356) : False (84.1%)
    2.1.2 Assets > 27,441 T USD (71)
      2.1.2.1 Return on equity <= -0.228 (7) : False (73.1%)
      2.1.2.2 Return on equity > -0.228 (64) : True (83.5%)
  2.2 Venture cerification = yes (175)
    2.2.1 Application area = Public area (52) : True (100%)
    2.2.2 Application area = Industry area (123)
      2.2.2.1 Industry = Manufacture of electronic components, computer, Manufacture of other machinery and equipment, Manufacture of chemicals and chemical products (89)
        2.2.2.1.1 Average debt ratio <= 1.225 (45)
          2.2.2.1.1.1 Ratio of subsidy <= 0.750 (23) : True (100%)
          2.2.2.1.1.2 Ratio of subsidy > 0.750 (22) : False (72.7%)
        2.2.2.1.2 Average debt ratio > 1.225 (44) : False (81.8%)
      2.2.2.2 Industry = Others (34) : True (100%)

Supplementary Material D2 A prediction rule for commercialization success by ML (C5.0)

1 Innobiz certification = yes (810)
  1.1 Firm age <= 20.5 years (736) : True (75.0%)
  1.2 Firm age > 20.5 years (74)
    1.2.1 Research area = Bio & Medical, Energy, ICT (10) : False (100%)
    1.2.2 Research area = Others (64)
      1.2.2.1 Average debt ratio <= 1.688 (53) : True (69.8%)
      1.2.2.2 Average debt ratio > 1.688 (11) : False (81.8%)
2 Innobiz certification = no (961)
  2.1 Venture certification = yes (183)
    2.1.1 Ratio of cash > 0.881 (172) : True (77.3%)
    2.1.2 Ratio of cash <= 0.881 (11) : False (72.7%)
  2.2 Venture certificaton = no (778)
    2.2.1 Research period <= 1.5 years (222) : False (54.1%)
    2.2.2 Research period > 1.5 years (556)
      2.2.2.1 Located in metropolitan = no (277) : True (64.3%)
      2.2.2.2 Located in metropolitan = yes (279)
        2.2.2.2.1 Ratio of budget to sales <= 0.569 (182)
          2.2.2.2.1.1 Research area = Chemistry (30)
            2.2.2.2.1.1.1 Average debt ratio <= 1.582 (19) : True (78.9%)
            2.2.2.2.1.1.2 Average debt ratio > 1.582 (11) : False (81.8%)
          2.2.2.2.1.2 Research area = Others (152) : False (60.0%)
        2.2.2.2.2 Ratio of budget to sales > 0.569 (97)
          2.2.2.2.2.1 Ratio of subsidy <= 0.830 (79) : True (77.2%)
          2.2.2.2.2.2 Ratio of subsidy > 0.830 (18) : False (66.7%)

Supplementary Material D3 A prediction rule for patent applications by ML (C5.0)

1 Ratio of subsidy > 0.873 (84) : False (76.2%)
2 Ratio of subsidy <= 0.873 (1,172)
  2.1 Research period <= 1.5 years (217)
    2.1.1 Venture cerification = yes (78)
      2.1.1.1 Debt ratio <= 3.224 (69) : True (58.3%)
      2.1.1.2 Debt ratio > 3.224 (9) : False (91.0%)
    2.1.2 Venture cerification = no (139) : False (72.0%)
  2.2 Research period > 1.5 years (955)
    2.2.1 Innobiz cerification = yes (474)
      2.2.1.1 Research area = Bio & Medical (123) : True (78.9%)
      2.2.1.2 Research area = Others (351)
        2.2.1.2.1 Located in metropolitan = no (165)
          2.2.1.2.1.1 Research area = Machinary , Energy, Chemistry, Knowledge services (123) : True (76.9%)
          2.2.1.2.1.2 Research area = Electronics (24) : False (66.7%)
          2.2.1.2.1.3 Research area = ICT (18)
            2.2.1.2.1.3.1 R&D investments <= 257.8 T USD (12) : False (85.9%)
            2.2.1.2.1.3.2 R&D investments > 257.8 T USD (6) : True (100%)
        2.2.1.2.2 Located in metropolitan = yes (186)
          2.2.1.2.2.1 Venture cerification = yes (109) : True (86.5%)
          2.2.1.2.2.2 Venture cerification = no (77)
            2.2.1.2.2.2.1 Ratio of MS & Ph.D. researchers <= 0.194 (23)
              2.2.1.2.2.2.1.1 Average debt ratio <= 1.127 (12) : True (75.0%)
              2.2.1.2.2.2.1.2 Average debt ratio > 1.127 (11) : False (100%)
            2.2.1.2.2.2.2 Ratio of MS & Ph.D. researchers > 0.194 (54) : True (73.5%)
    2.2.2 Innobiz cerification = no (481)
      2.2.2.1 Research area = Machinary, Knowledge services (144) : False (57.6%)
      2.2.2.2 Research area = Bio & Medical, Energy, ICT, Chemistry (262) : True (57.3%)
      2.2.2.3 Research area = Electronics (75)
        2.2.2.3.1 Average total asset turnover <= 1.265 (66)
          2.2.2.3.1.1 Number of CR <= 1 (40) : False (69.9%)
          2.2.2.3.1.2 Number of CR > 1 (26)
            2.2.2.3.1.2.1 Ratio of female researchers <= 0.082 (11) : False (81.8%)
            2.2.2.3.1.2.2 Ratio of female researchers > 0.082 (15) : True (93.3%)
        2.2.2.3.2 Average total asset turnover > 1.265 (9) : True (98.7%)

Supplementary Material E The prediction models by logistic regression (LR)

Although the linear models showed somewhat inferior classification performance, they provided more concise and statistically significant rules. The variables selected based on the statistical significance for each performance indicator are well included in the key factors by ML. In addition, each rule by LR can be theoretically explained in connection with the results of preceding studies.

Table SE 1. The prediction models by logistic regression (LR)

Factors	R&D success			Commercialization			Patent applications		
	B	S.E.	Exp(B)	B	S.E.	Exp(B)	B	S.E.	Exp(B)
Innobiz certification	1.10^{***}	0.17	0.33	0.52^{***}	0.12	0.60	0.36^{**}	0.11	0.70
Venture certification	0.97^{*}	0.17	0.38	0.31^{**}	0.12	0.73	0.23^{*}	0.11	0.80
Research period	0.91^{***}	0.17	2.49	0.47^{***}	0.12	1.60	0.66^{***}	0.12	1.93
Ratio of MS & Ph.D. researchers	0.60^{*}	0.28	1.82				0.47^{*}	0.19	1.59
Average debt ratio	-0.30^{*}	0.13	0.74						
Ratio of cash	2.97^{*}	1.15	19.40						
Ratio of researchers with degrees in Eng.	0.63^{*}	0.26	1.88						
Assets	$1.2E\text{-}5^{**}$	4.0E-6	1.00						
Firm age				-0.02^{**}	0.01	0.98			
Ratio of female researchers							0.77^{*}	0.31	2.15
Constant	-3.03^{*}	1.18	-	0.54^{*}	0.24	-	-1.01^{***}	0.24	-
n	1,771			1,771			1,771		
χ^2	234.83^{***}			73.74^{***}			84.76^{***}		
-2Log-likelihood	1,241.29			2,215.97			2,365.49		
Pseudo R^2	0.26			0.06			0.06		

$^{*}p < 0.05$, $^{**}p < 0.01$, $^{***}p < 0.001$

In the model for R&D success, R&D projects that included a higher ratio of researchers with degrees in engineering and with graduate degrees (MS & Ph.D.) had a higher probability of R&D success. R&D is an innovative task that requires a knowledge-intensive perspective and creativity[1]. Most of the R&D projects sponsored by the program are concentrated in fields of engineering, and therefore it is understandable that the participation of experts related to these fields will have an important influence on achieving the R&D performance goals. In addition, compared to projects with a research period of 1 year, projects with relatively sufficient time (2 years) were more likely to achieve R&D success. Regarding the composition of research budget, it can be understood that the higher the ratio of cash with a high degree of freedom in use, the more effective research is promoted, which contributes to R&D success. Regarding the firm attributes, firms that were pre-certified by venture capital or public agencies as having innovative potential and capacity were more likely to achieve R&D success. In addition, a firm's assets can act as an important resource and capability to continuously and stably carry out R&D. Meanwhile, firms that belong to an industry with a high average debt ratio, resulting in lower financial stability, were notably found to have relative weak likelihood of R&D success.

In terms of commercialization, firms that received venture and Innobiz certifications showed a higher likelihood of success. Sufficient research period was found to have a positive effect on commercialization as well. Meanwhile, firm age was found to be a negative factor affecting commercialization. It can be attributed to the fact that firms that have operated well for at least a certain number of years tend to have a significant proportion of its production capabilities already devoted to an existing flagship product, which may delay the timing of input for new products and delay sales [2].

Table SE1 showed that patent application was significantly affected by factors related to personnel composition. We found that that a higher ratio of researchers with MS & Ph.D. degrees and a higher ratio of female researchers raised the possibility of patent applications. To apply a patent, it is necessary to draw on in-depth knowledge of cutting-edge technologies to persuasively demonstrate novelty and progress, and therefore, participating researchers with longer years of experiences in related fields will increase the possibility of achieving patents[3]. Gender diversity in R&D teams could promote innovation efficiency and innovation performances by providing various ways of thinking, perspectives, and attitudes[4]. The ratio of female researchers increases gender diversity, as about 85% of the researchers who participated in the program were male. We believe that gender diversity can generate significant informational and social benefits[5], and the positive effects increased patent applications. Also, as in other performance indicators, venture certification, Innobiz certification, and research period were found to have a positive effect on patent applications.

References

1 Zouaghi, F., Garcia-Marco, T. & Martinez, M. G. The link between R&D team diversity and innovative performance: A mediated moderation model. Technological Forecasting and Social Change 161, 120325 (2020).

2 Arrow, K. Economic welfare and the allocation of resources for invention. The rate and direction of inventive activity: economic and social factors. N. Bureau (1962).

3 Nagesh, D. & Thomas, S. Success factors of public funded R&D projects. Current Science, 357-363 (2015).

4 Xie, L., Zhou, J., Zong, Q. & Lu, Q. Gender diversity in R&D teams and innovation efficiency: Role of the innovation context☆. Research Policy 49, 103885 (2020).

5 Cox, T. H., Lobel, S. A. & McLeod, P. L. Effects of ethnic group cultural differences on cooperative and competitive behavior on a group task. Academy of management journal 34, 827-847 (1991).
